# Synthesis, Characterization, and Assessment of a CeO_2_@Nanoclay Nanocomposite for Enhanced Oil Recovery

**DOI:** 10.3390/nano10112280

**Published:** 2020-11-17

**Authors:** Mohammad Javad Nazarahari, Abbas Khaksar Manshad, Siyamak Moradi, Ali Shafiei, Jagar Abdulazez Ali, S. Mohammad Sajadi, Alireza Keshavarz

**Affiliations:** 1 Department of Petroleum Engineering, Abadan Faculty of Petroleum Engineering, Petroleum University of Technology (PUT), Abadan 63187-14317, Iran; mohammadnazarahari@yahoo.com (M.J.N.); moradi.s@put.ac.ir (S.M.); 2 Petroleum Engineering Program, School of Mining & Geosciences, Nazarbayev University, Nur-Sultan 010000, Kazakhstan; 3 Department of Petroleum Engineering, Faculty of Engineering, Soran University, Kurdistan Regional Government, Soran P.O. Box 624, Iraq; jagar.ali@soran.edu.iq; 4 Department of Nutrition, College of Health Technology, Cihan University-Erbil, Kurdistan Regional Government, Erbil P.O. Box 623, Iraq; smohammad.sajadi@gmail.com; 5 School of Engineering, Edith Cowan University, 270 Joondalup Drive, Joondalup 6027, Australia; a.keshavarz@ecu.edu.au

**Keywords:** clay nanocomposite, enhanced oil recovery, nanocomposite, synthesis, characterization, nanofluid, interfacial tension, wettability alteration, carbonate reservoirs, sandstone reservoirs

## Abstract

In this paper, synthesis and characterization of a novel CeO_2_/nanoclay nanocomposite (NC) and its effects on IFT reduction and wettability alteration is reported in the literature for the first time. The NC was characterized using scanning electron microscopy (SEM), X-ray diffraction (XRD), Fourier-transform infrared spectroscopy (FTIR), thermogravimetric analysis (TGA), energy-dispersive X-ray spectroscopy (EDS), and EDS MAP. The surface morphology, crystalline phases, and functional groups of the novel NC were investigated. Nanofluids with different concentrations of 100, 250, 500, 1000, 1500, and 2000 ppm were prepared and used as dispersants in porous media. The stability, pH, conductivity, IFT, and wettability alternation characteristics of the prepared nanofluids were examined to find out the optimum concentration for the selected carbonate and sandstone reservoir rocks. Conductivity and zeta potential measurements showed that a nanofluid with concentration of 500 ppm can reduce the IFT from 35 mN/m to 17 mN/m (48.5% reduction) and alter the contact angle of the tested carbonate and sandstone reservoir rock samples from 139° to 53° (38% improvement in wettability alteration) and 123° to 90° (27% improvement in wettability alteration), respectively. A cubic fluorite structure was identified for CeO_2_ using the standard XRD data. FESEM revealed that the surface morphology of the NC has a layer sheet morphology of CeO_2_/SiO_2_ nanocomposite and the particle sizes are approximately 20 to 26 nm. TGA analysis results shows that the novel NC has a high stability at 90 °C which is a typical upper bound temperature in petroleum reservoirs. Zeta potential peaks at concentration of 500 ppm which is a sign of stabilty of the nanofluid. The results of this study can be used in design of optimum yet effective EOR schemes for both carbobate and sandstone petroleum reservoirs.

## 1. Introduction

The global demand for energy resources is increasing steadily due to the world population growth and higher energy demands from developing economies. Petroleum will make up a notable part of the energy consumption in the decades to come, 40–60% in different scenario, despite the fact that production from existing reservoirs is declining rapidly over time and new discoveries are not able to close the gap [1]. The residual oil trapped in the rock and the resistant forces prevent further petroleum production at desired rates. Well over 50% of the oil in place in reservoirs is trapped in pores and cannot be produced through natural depletion. After primary stage of production, water and gas injection practice is common for pressure maintenance and enhanced oil recvery [2]. On average, the cumulative recovery factor (RF) during both primary and secondary stages of oil recovery does not exceed 30% [3]. Hence, application of more effective and efficient enhanced oil recovery (EOR) processes is inevitable [4,5].

Enhanced oil recovery (EOR) processes focus on overcoming these preventive and advective phenomena to enhance the petroleum recovery and increase the cumulative recovery factor from the reservoir. A process involving injection of fluids into a petroleum reservoir to overcome resistant forces by way of interacting with rock-oil-water system to improve the petroleum production is known as an enhanced oil recovery or EOR process [3]. Application of EOR techniques can potentially increase the ultimate RF by 30–60%. EOR methods can be classified into three major classes: chemical, gas injection, and thermal methods. Depending on the EOR method used, they can enhance the oil recovery once introduced to a reservoir by reducing the oil viscosity, and altering viscous forces and the bond between oil-water-rock facilitating the mobility of the residual oil [6,7].

Application of nanoparticles (NPs) and nanocomposites (NCs) to enhance oil production in various EOR methods has been a subject of interest for researchers [8,9]. Polymers, surfactants, NPs, NCs, or a combination of these materials is commonly used in chemical EOR methods to enhance the incremental oil recovery [10]. Adding chemical agents such as polymers can help to improve the performance of the EOR processes via increasing the water viscosity to resolve the mobility ration issue and enhance the macroscopic displacement by adding the polymer to the injection solution into the reservoir [11]. Injection of surfactant can reduce the interfacial tension (IFT) between the water and oil which can improve the microscopic displacement during the EOR process [12]. Surfactants can also affect the wettability of the reservoir rock towards a more water-wet state, which can lead to increase in oil production [13,14,15]. In addition, nanoparticles used in EOR process are environmental friendly and can reach the reservoir rock better because of their small size [16].

NPs used in EOR process can be classified into four groups: natural, inorganic, metal oxide, and magnetic [9]. NPs are used for preparation of nanofluids, nanoemulsions, and nanocatalysts used in EOR processes [17]. Nanofluids contain at least one added material of less than 100 nm in size and are used as a base fluid in EOR processes [9]. Nanoemulsions used in EOR processes contain materials of 50–500 nm in size [9]. However, they are usually larger than 100 nm in size. Nanocatalysts are typically used to reduce the oil viscosity during steam injection processes (i.e., thermal oil recovery commonly used for extraction of heavy, extra heavy oil, and bitumen) [18]. The particle size is essential in chemical EOR processed because particles may block the pore space and reduce porosity and permeability of the reservoir. However, thanks to very small size of NPs which is on the range of 1–100 nm, injecting them as an additive to chemical EOR processes does not impair the reservoir porosity and permeability and at the same time can assist in mobilizing and displacing the residual oil [9]. Nanocatalysts have a higher contact surface than conventional catalysts [19]. This implies that the NPs assisted chemical EOR process leading to an increased oil recovery can reach a much bigger area of the reservoir [17]. The cost of NPs is also less than chemical materials. Hence, adding NPs to chemical EOR processes and further optimizing the process can reduce the cost of production of the EOR oil [20,21].

Application of NPs and NCs to enhance and optimize oil recovery processes has been a subject of research interest in the past few decades. Several scientists have investigated the mechanisms by which NPs and NCs assist in increasing oil recovery. NPs can affect the reservoir rock and fluid via alteration of oil viscosity, IFT, reservoir rock wettability, electrostatic forces, and control of fines migration [18,22,23,24,25,26]. Some of the mechanisms are reviewed and summarized in Table 1. Application of CeO_2_ can reduce the IFT and change the viscosity. But, it has a samll effect on wettability alteration [9,27]. Nanoclays due to their layered structure and rheological properties can affect the wettability alteration and viscosity [28,29].

Synthesis and application of environmentally green nanocomposites with applications for various purposes including EOR and developing drilling muds has been a subject of research interest for researchers. In a recent study, researchers introduced a green nanocomposite synthesized using combination of cellulose nanocrystals with graphene-based materials. The nanocomposite showed excellent physical and chemical characteristics. However, the researchers did not use it for EOR. Such nano-materials have potential to be used EOR agents. This is require further research to understand the performance of the new nano-material for EOR purposes [41]. In another recent publication, researchers provided a very comprehensive review of chemistry, structure, and applications of nanocomposite from biorenewable sources. This work can serve as a good basis for EOR scientists for synthesis, characterization, and application of novel and existing bio-nanocomposites as environmental friendly chemical EOR agents [42].

Several studies on application of various nanoparticles combined with polymers and surfactnats as chemical additivies for EOR processes are reported in the literature. Researchers have investigated the the feasibility of EOR by using core-shell polymeric nanoparticles suspension in a low permeability heterogeneous oil reservoir [43]; conducted some high temperature (120 °C) corefloods to study the feasibility of using cellulose nanocrystals as additives to enhance the oil recovery of EOR processes [44]; used coreflooding and microfluidics to assesse the possibility of application of nanocellulose as an additive to enhance the efficiency of waterflooding [45]; conducted secondary and tertiary corefloods with polymer-coated silica nanoparticles on neutral-wet Berea sandstine samples saturated with a North Sea crude oil under ambient conditions and evaluated the factors affecting oil recovery [46]; reported experimental and filed scale research on design and application of nanofluid for EOR purposes aiming at understandibg the interactions between surfactant-nanoparticle and brine [47]; studied silica nanoparticles’ stability under reservoir conditions for EOR purposes [48]; investigated the relations between pore size distribution and oil-water relative permeability on effectivenese of anionic surfactant and silica nanoparticles in EOR processes in carbonate petroleum reservoirs. Researchers have recently investigated the possibility of coupling conventional nanofluid flooding with electromagnetic waves of varying frequencies to boost the oil mobility in the porous media and alter the oil-nanoparticle interface to facilitate detachment of oil droplets from the rock surface and to encourage their flow into the production well. The experimental investigation involved conducting displacement tests in water-wet sandpacks at 95 °C using alumina nanofluids in four different transition phases and particle sizes ranging from 25 to 94.3 nm. A crude oil obtained from Tapis (Malaysia), was used in the study. The electromagnetic waves assisted nanofluid flooding proved more effective compared with the conventional nanofluid flooding (i.e., no electromagnetic waves) by improving the oil recovery factor by additional 4.12 to 12.9% of the OOIP. The researchers concluded that the hybrid EOR process is frequency dependent. They also have attributed the observed flow enhancement to an electrorheological effect induced by the electromagnetic waves applied and disturbances in the oil-nanoparticle interface caused by deformation of the oil droplet [49].

Very few stuide are also reported in the literature on potential application of nanoparticles in processes related to thermal EOR. An optimization study is reported on use of CeO_2_ nanoparticles with different metal oxides to maximize catalytic steam decomposition of asphaltenes at low temperature to investigate the effects of using nanopartciles on improving the efficiency of steam injection as a thermal EOR method for recovery of heavy oil and bitumen [48]. Regenerating impacts of NiO-PdO/CeO_2±δ_ nanocomposite on adsorption behaviour and decomposition of n-C_7_ asphaltenes during steam gasification processes was also investigated by some researchers. The asphaltenes exhibited high adsorption affinity to the surface of the nanoparticles despite a slight reduction in the catalytic activity and adsorption capacity of the nanoparticles.

Studies on the application of nanoparticles for enhancement of conformance conrol in EOR operations has been reported in the literature as well. The effect of nanoparticles with various chemistry on stability and rheology of acrylamide sodium acrylate copolymer/chromium (III) acetate gel for the purpsoe of conformance control in EOR operations were investigated [50]. Synthesis of multipurpose terpolymers based on styrene, *tert*-butyl methacrylate, and glycidyl methacrylate and their modification non-covalent functionalization of carbon nanotubes is reported in the literature. Some of the polymers showed a good thickening properties with potential applications in water rheology control for EOR purposes (i.e., waterflooding in heavy crude oils) [51].

Some fundamental researches were also reported in the literature on investigatin of behaviour of nanomaterials in porous media and exploring the mechanisms involved in EOR processes. The transport behavior and retention of nanocellulose in porous media, sandpacks and Berea sandstone cores, as a potentail chemical additve to some EOR processes (i.e., waterflooding) was investigated. The researchers concluded that during low salinity waterflooding, adsorption was the dominant retention mechanism. Log-jamming was reported to be the dominant retention mechanism as the water salinity increased duing the experiments. Salinity was identified as the major factor affecting transport properties. Higher flow rates also were found effective to lessen both retention and permeability reduction [52]. Micromodels were used to visually study the impacts of using nanopartciles on performance of polymer flooding in water wet porous media. They concluded that the biopolymer content did not help nanoparticles to reduce the IFT. In addition, a homogenous dispersion of the nanoparticles in the solution and a reduction in the polymer adsorption was observed that can explain the enhancement in the sweep eciency and oil recovery [53].

To the best of our knowledge, synthesis and caracterization of a CeO_2_@nanoclay NC to combine the advantages of both CeO_2_ (in reducing the IFT and viscosity) and nanoclays (in altering the wettability) to enhance and optimize EOR processes is not reported in the literature yet. Biosynthesis and characterization of the novel CeO_2_/nanoclay NC and its effects on IFT reduction and wettability alteration using state-of-ther-art laboaratory facilities such as scanning electron microscopy (SEM), X-ray diffraction (XRD), Fourier-transform infrared spectroscopy (FTIR), thermogravimetric analysis (TGA), energy-dispersive X-ray spectroscopy (EDS), and EDS MAP is being reported in the literature for the first time.

This paper is organized in two parts. In the first part, synthesis and characterization of a novel CeO_2_@Nanoclay by using the state-of-the-art laboratory facilities listed above is presented. In the second part, different concentrations of the novel nanoclay nanocomposite was used to prepare nanofluids with different concentrations of 100, 250, 500, 1000, 1500, and 2000 ppm. Then, stability, pH, conductivity, IFT, and wettability alternation characteristics of the nanofluids as factors affeting performance of EOR processes were tested to determine optimum concentration of the NC for EOR purposes in the selected carbonate and sandstone reservoir rock samples. The results obtained from this study then are discussed in detail. The outcome of this study can be used in design of optimum and effective EOR schemes for both carbobate and sandstone petroleum reservoirs.

## 2. Materials and Methods

In this section the materials used in this study such as crude oil and reservoir rock samples are described.

### 2.1. Materials

#### 2.1.1. Crude Oil

The crude oil used in his study is a dead crude oil collected from the Gachsaran reservoir in SW Iran. The crude oil has an acidity of 3.7 mg KOH/g, density of 0.84 g/cm^3^ and the viscosity of 40 cP. The results from the analysis of the dead oil are presented in Table 2.

#### 2.1.2. Reservoir Rock Samples

In this study, two reservoir rock types were used in this research work: 1- a carbonate rock obtained from the Asmari Carbonate Formation outcrop in Southwestern Iran. This carbonate rock contains 95% carbonate calcium. 2- a sandstone reservoir rock obtained from the Aghajari Formation outcrop in Southwestern Iran. The rock contains 63% SiO_2_ and 37% CaCO_3_.

#### 2.1.3. Aqueous Phase

Distilled water was used to prepare the nanofluid which has a density of 0.998 g/cm^3^. The electrical conductivity of the distilled water is 38–41 μs/cm and has a pH of 7.02.

### 2.2. Synthesis of CeO_2_@Nanoclay Nanocomposite

In this research work, we synthesized and used a novel nanocomposite (CeO_2_@nanoclay) The method of preparation and synthesis of this material is as follows: *Capsicum frutescent* from the Solanaceae family is a genus of flowering plant that widely cultivated in South East Asia and South America [54]. The plant is a rich source of antioxidants and commonly is used as a spice, and as an antibacterial, antimicrobial, and antibiotic herb [55]. The plant extract is enriched by many valuable antioxidant and other types of phytochemicals such as vitamins A, E, C, K, B6, folic acid, niacin, and caffeic acid, caproic acid, capsaicin, dihydrocapsaicin, cinnamic acid, para-coumaric acid, ferulic acid, mevalonic acid, pyrazine derivative, calcidiol, kaempferol derivative, quercetin derivative, and lipids. Thus, concerning this valuable content of phytochemicals, we decided to use the plant extract as a green media containing reducing, stabilizing, and capping agents [56,57].

#### 2.2.1. Preparation of the Plant Extract

First, 50 g of dried powder of the *Capsicum frutescent* leaves was mixed with 400 mL of distilled water at 80 °C for 30 min. Then, the aqueous extract was filtered and stored at 4 °C for further use in the experiment.

#### 2.2.2. Synthesis of CeO_2_@Nanoclay NCs

**Scheme 1:**
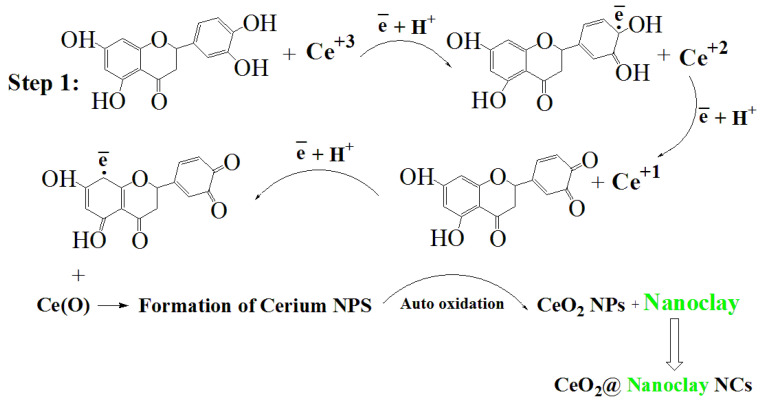
Schematic of a possible mechanism for synthesis of CeO_2_@nanoclay nanocomposite.

First, 2 g of Ce(OH)_3_ was mixed with 100 mL of the *Capsicum frutescent* extract; then, 10 g of nanoclay was added to the mixture while stirring at 80 °C for 10 h at pH of 9. The particles precipitated at the bottom of the container were separated, dried, and kept for use for the experiments. A possible chemical reaction mechanisms for the synthesized nanocomposute in presented in Scheme 1.

### 2.3. Nanofluid Preparation

To prepare nanofluids with concentration of 100, 250, 500, 1000, 1500, and 2000 ppm, the required amount of nanocomposite was measured using a digital scale and then homogenized using N ultrasonic disperser and vigorous magnetic agitation. Then deionized water (DW) was added to the mix and was stirred for 1 h at 70 °C by using a magnetic stirrer ensure complete dissolution of the particles in the solution. Then the solution was placed into an ultrasonic device (200 W UP200H, Hielscher, Teltow, Germany) for 1.5 h to increase the stability of the solution for testing. Afterward, nanofluid solutions were prepared at different concentrations and their density was measured by suing a densitometer (KEM DA-650 Density/Specific Kyoto Electronics, Tokyo, Japan). pH tests were also conducted with a pH meeter to measure acidity of the solutions (Mettler Toledo, Columbus, OH, USA). Conductivity measurements were also carried out to determine salinity of the solutions, their ionic strength, and electrical resistance of each solution (model cond7310, WIW Company, Weilheim, Germany). In the final step, zeta potential measurements were done to assess the stability of nanofluid that is considered as an important parameter in the laboratory investigation of EOR process (Zetasizer Nano ZS90, Malvern, UK).

## 3. Evaluation of the Nanofluids

In this section, some properties of the prepared nanofluids such as interfacial tension (IFT) which are very important in performance assessment of EOR processes are measured.

### 3.1. IFT Measurement

**Figure 1:**
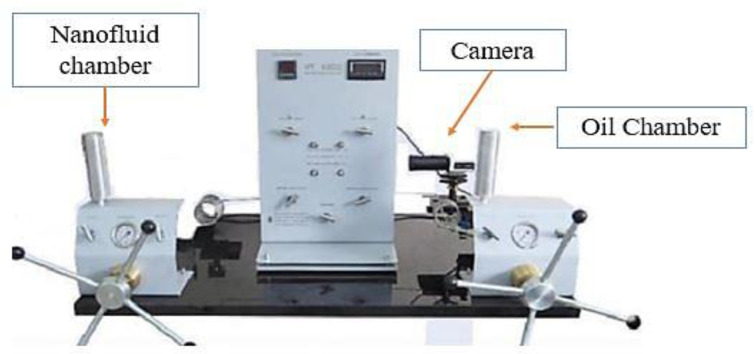
The VIT-6000 apparatus used in this research work for IFT and contact angle measurements. This device used an online image capturing system for recording data under a given pressure and temperature, periodically.

Interfacial tension or IFT plays an important role in performance of many EOR process involving injection of chemicals into an oil reservoir. Hence, the effect of injection of chemical agents on IFT should be investigated in laboratory. In this research work, IFT measurement tests were conducted using a VIT-6000 system (EOR Fars, Shiraz, Iran). The setup is designed to measure IFT by using the pendant drop method for liquid-liquid and liquid-gas (drop) at pressures and temperatures up to 400 bars and 150 °C, respectively (Figure 1). The apparatus can measure both IFT and contact angle using an online image capturing system capable of recording data under a given pressure and temperature, periodically. The test is conducted via injection of a drop into a bulk phase under a given pressure and temperature. For contact angle measurement, the drop is placed on a plane solid sample. Before the pendant drop test, the VIT-6000 device is cleaned. The VIT-6000 is calibrated with IFT measurement between the toluene and water that is about 30 mN/m.

First nanofluid solution and crude oil are prepared. Then, their solution is poured into a solution injection chamber and the crude oil is poured into another injection chamber. The brine or nanofluid is pumped from the injection chamber into the main chamber of the device. The main chamber fills with brine or nanofluid. The crude oil is injected from the injection chamber into the needle device; then, a drop of the crude oil on the needle of the device is hung to the brine or nanofluid solution. The camera captures and sends the image of the crude oil drop inside the brine or nanofluid to the computer. Then, the software processes the image and determined the IFT value.

### 3.2. Contact Angle Measurement

Reservoir rock wettability plays a pivotal role in oil displacement process through porous media. The pore space in a petroleum reservoir is filled with more than one fluid where each has a different surface adherence tendency. Hence, there is a competitive condition where different fluids stick to the rock surface with different intensities. Therefore, each makes a different contact angle with the rock surface that is a measure of wettability of the rock surface. Wettability alteration in a petroleum reservoir from oil-wet towards water-wet is one of the main mechanisms in petroleum production of oil by using nanofluids in both carbonate and sandstone reservoirs [18].

**Figure 2:**
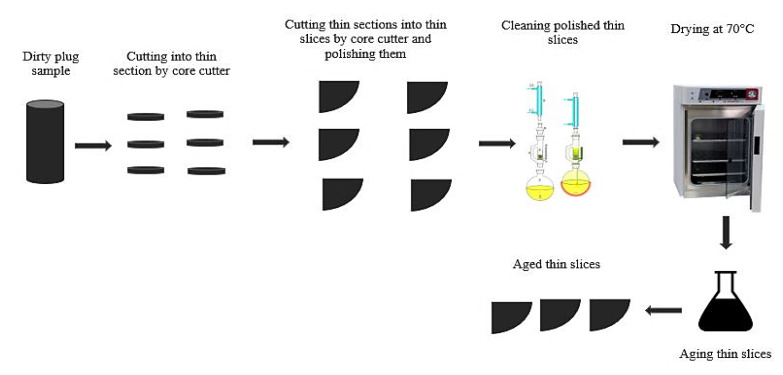
A schematic of the thin section rock aging setup and the workflow used for preparation of rock samples for contact angle measurement.

In this section, the effect of different prepared nanofluid solutions on wettability alteration of the reservoir rocks tested in this study are investigated. Contact angle measurement tests were conducted at room temperature. For this purpose, first some oil-wet thin sections were prepared. This was achieved by placing the thin sections into a crude oil container and then placing the container in the oven at 80 °C for 7 days to become completely oil-wet (Figure 2). In the next step, the thin sections were removed from the container, rinsed with kerosene, and dried. Then, the thin sections were placed in a container and the container was filled with kerosene, and nanofluid solution for 48 h. The droplets were dropping from the needle and were placed on the surface of the thin section. At the beginning of the test, the contact angle was measured in presence of all nanofluids. The purpose was to determine the best concentration of nanofluid.

## 4. Results and Discussion

In this section, the results obtined from this experimental reserch work are presented and discussed. First, different characteristics of the synthesized novel CeO_2_@nanoclay such as surface morphology, crystalline phases, and functional groups are described and discussed using data obtaoined from various state of the art analytical methods used such as XRD, FTIR, FESEM, TGA, DES, and EDX MAP. Then, various properties of the nanofluids prepared by using different concentratioins of the novel CeO_2_@nanoclay such as density, pH, viscosity, conductivuty, IFT, zeta potential, and contact angle along with their effect on IFT and wettability charateristics of the stuided reservoir rocks are presented and discussed. The later section aims at understanding the effects of using the novel CeO_2_@nanoclay on improvent of oil recovery from both sandstone and carbonate reservoirs via enhancement of oil production through reduction in IFT, oil viscosity, and contact angle (i.e., wettability alteraion towards a more water wet state).

### 4.1. Nanocomposite Characterization

To characterize the novel nanocomposite, some of its properties including surface morphology, crystalline phases, and functional groups were characterized using X-ray diffraction (XRD), Fourier-transform infrared spectroscopy (FTIR), field emission scanning electron microscopy (FESEM), thermogravimetric analysis (TGA), and energy-dispersive X-ray spectroscopy EDS and EDX MAP. The results obtained from this part of the study are presented and discussed in the consequent sub-sections.

#### 4.1.1. X-ray Diffraction Characterization Test (XRD)

**Figure 3:**
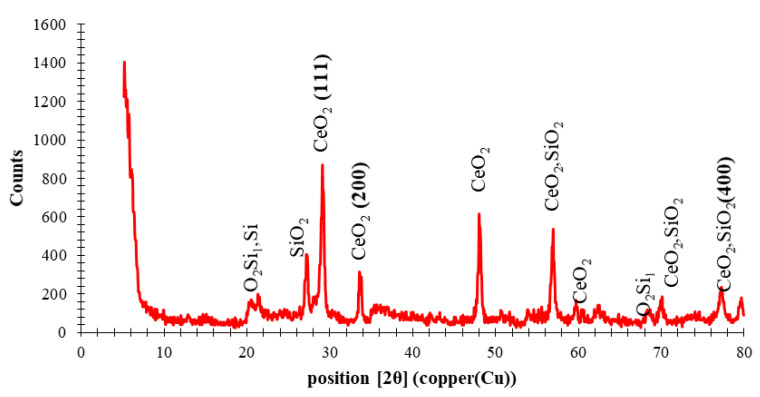
The XRD graph of the nanocomposite used for precise determination of the crystalline phase and size of the nanocomposite. The morphology of CeO_2_@Nanoclay nanocomposite is calcined. CeO_2_ is the predominant phase based on the diffraction patterns of CeO_2_ nanoparticles with different patterns of SiO_2_.

The XRD test (Figure 3) was carried out for precise determination of crystalline phase and size of the nanocomposite. The morphology of CeO_2_@nanoclay nanocomposite is calcined. As shown in Figure 3, diffraction patterns of CeO_2_ nanoparticles with different patterns of SiO_2_ phase shows that CeO_2_ is the predominant phase. The governed peaks at angles of 29.15°, 48.05°, and 56.9° are related to CeO_2_ crystallography planes of (111), (220), and (311), respectively. A cubic fluorite structure was identified for CeO_2_ using the standard data. The mean size of the ordered CeO_2_ nanoparticles was estimated from the full width at half maximum (FWHM) using a relationship proposed by Debye Sherrer [7]:

D = 0.89λ/Bcos θ(1)

where 0.89 is the shape factor, λ is the x-ray wavelength, B is the line broadening at half the maximum intensity (FWHM) in radians, and θ is the Bragg angle.

Based on Equation (1) and (111) crystallography plane of the CeO_2_, the diameter of the nanomaterials is calculated as 38.4 nm. A comparison of CeO_2_ peaks by reference peaks shows a little shifting to larger angles can be attributed to positioning of the CeO_2_ in the state of nanocomposite instead of nanoparticle form. Available peaks at the angle of 27.2° correspond to SiO_2_ and the particle size is calculated as 27.5 nm [31,58].

#### 4.1.2. Fourier-Transform Infrared Spectroscopy Characterization Test (FTIR)

**Figure 4:**
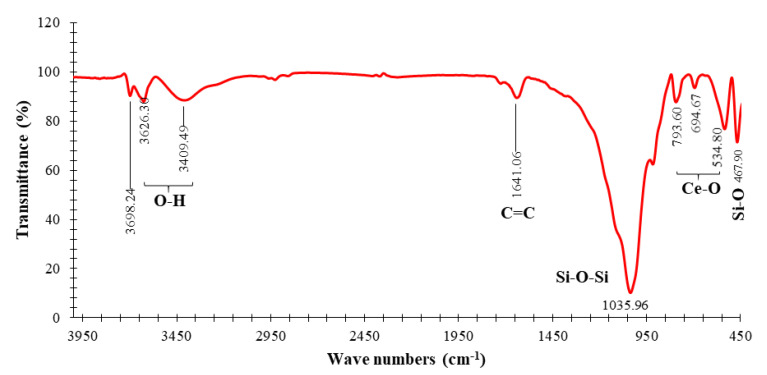
FTIR graph of the NC used for precise determination of chemical composition of the NC and identification of wave number, chemical bonds, and functional groups of the compound.

To determine the chemical composition of the nanocomposites more precisely, we used a FTIR test (Figure 4). As shown in Figure 4, the infrared spectrum (FTIR) of the synthesized NC (CeO_2_@nanoclay) was on the range of 400–4000 cm^−1^ wave number and the chemical bonds and functional groups of the compound were also identified. The large broad bands at 3409 cm^−1^ and 3626 cm^−1^ are attributed to the stretching vibration of O–H in OH groups. The absorption peaks of approximately 1641 cm^−1^ were attributed to the bending vibration of the C=C stretching. The spectrum of the sample and a broad peak at 1036 cm^−1^, 793.60 cm^−1^, and the small peak at 467 cm^−1^ corresponds to the Si–O–Si (Si–O bending vibration band and asymmetric stretching vibration of Si–O band). The strong band below 700 cm^−1^ was allocated to the Ce–O stretching mode. A broadband comparable to the Ce–O stretching mode of CeO_2_ is seen at 500 cm^−1^ [59].

#### 4.1.3. Field Emission Scanning Electron Microscopy Characterization Test (FESEM)

**Figure 5:**
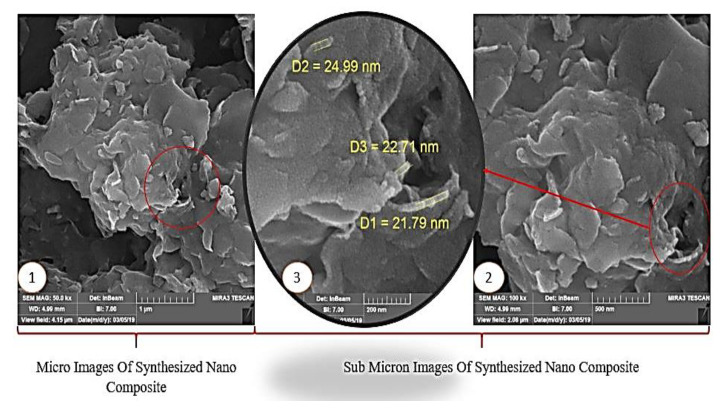
FESEM images from the NC showing a layer sheet surface morphology with particle sizes ranging from 20 to 26 nm.

Field emission scanning electron microscopy (FESEM) can help to determine the surface morphology of the synthesized nanocomposite. Layer sheet morphology of CeO_2_/SiO_2_ nanocomposite are shown in Figure 5. The particle sizes are approximately 20 to 26 nm that are slightly larger than the particle sizes predicted by Debye-Scherer’s (Equation (1)).

#### 4.1.4. Thermogravimetric Analysis (TGA)

**Figure 6:**
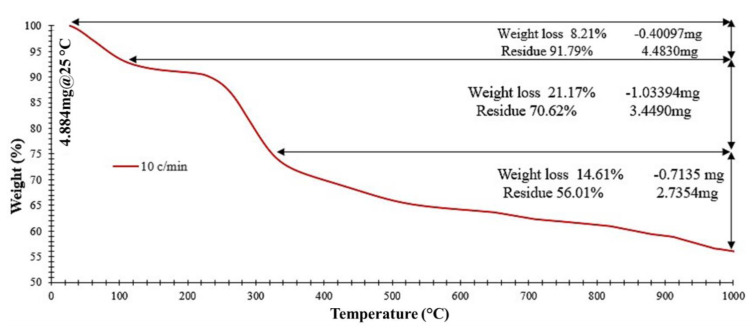
TGA analysis result used to investigate the thermal stability of the novel NC. The novel NC has a low weight loss of 8.21% and a high stability at 90 °C, which is a typical upper bound temperature in petroleum reservoirs.

The TGA analysis was used in this research work to determine the thermal stability and the effect of temperature on the stability of the nanocomposite. 4.884 mg of the synthesized nanocomposite was exposed to an environment containing argon. The rate of increase in temperature was about 10 °C/min. As shown in Figure 6, the nanocomposite shows several stages of weight loss with increase in temperature. The first stage of weight loss occurs at temperatures below 200 °C, which indicates the separation and vaporization of the surface water depending on the type of material structure. The second stage of weight loss takes place from 200 to 400 °C which may indicate the separation of inter-structural water and decomposition of cerium carbonate. And lastly, at temperatures above 400 °C. This weight loss can be attributed to burning structural water (organic chain) and conversion of water to CO. According to the results, this material shows a weight loss of 2.51 mg (44.04%) at 1000 °C. This nanocomposite exhibits low weight loss (8.21%) and high stability at 90 °C which is a typical upper bound temperature in petroleum reservoirs [60,61].

#### 4.1.5. Energy-Dispersive X-ray Spectroscopy (EDS) and EDX MAP

**Figure 7:**
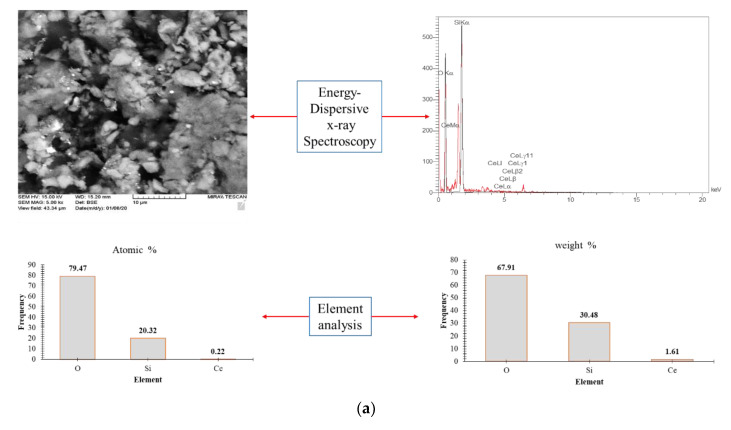

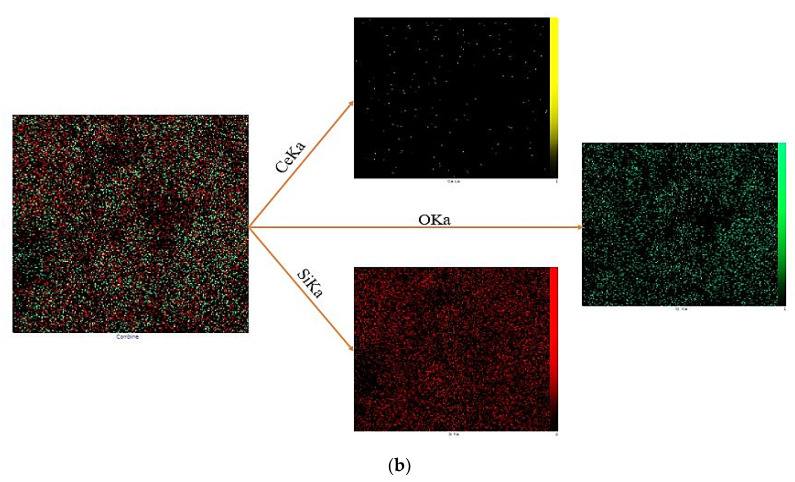
EDS and EDS-MAP analysis results which were used, respectively, in this research work to identify the constituent elements and the frequency distribution of the elements and the distribution and dispersion of each element found in a given area in the novel NC, quantitatively. (**a**) shows the presence of O, Ce, and Si in the novel NC. Atomic and weight analysis of O, Si, and Ce of the NC are determined as (79.47%, 20.32%, and 0.225%) and (67.91%, 30.48%, and 1.61%), respectively. (**b**) shows the distribution and dispersion of each O, Si, Ce in a given area in the NC based on the EDS-MAP, quantitatively.

EDS and EDS-MAP analysis were used in this research work to identify the constituent elements and the frequency distribution of the elements in the nanocomposite (Figure 7a,b). As shown in Figure 7a, the EDS analysis confirms the presence of O, Si, and Ce in the novel NC. Atomic and weight analysis of O, Si, and Ce of the NC are determined as (79.47%, 20.32%, and 0.225%) and (67.91%, 30.48%, and 1.61%), respectively. EDS-MAP of the NC is presented in Figure 7b. The EDS-MAP shows the distribution and dispersion of each O, Si, and Ce in a given area in the NC, quantitatively.

### 4.2. Nanofluid Properties

To evaluate the effectiveness and success of a substance used in EOR processes, the performance of this substance in all stages of design, evaluation, and implementation should be examined and optimized in a comprehensive way. It is essential to optimize the IFT and contact angle during the EOR process as any changes in these parameters can greatly impact the oil recovery.

In this research work, to understand the effects of the novel CeO_2_@nanoclay on improvent of oil recovery from both sandstone and carbonate reservoirs different characteristics of the nanofluids prepared by using different concentratioins of the novel CeO_2_@nanoclay such as density, pH, viscosity, conductivity, IFT, zeta potential, and contact angle along with their effect on IFT and wettability charateristics of the stuided reservoir rocks needs to be examined. Insights obtained from characterization of the prepared nanofluids sets the foundation for quantitative assessemnt of the effects of using the novel CeO_2_@nanoclay as an additive in the form of a nanofluid on enhancement of oil production through well-known mechanisms govering performance of EOR processes such as reduction in IFT, oil viscosity, and contact angle (i.e., wettability alteraion towards a more water wet state) throuh the interactions between the rock and fluids in the reservoir. The out come of such complex interactions can potentially lead to unlocking more oil from the pore space and imrpving the production rate and the recovery factor (RF) of the petroleum reservoir. The tests results are presented in the Table 3 and then are described and discussed in the cosequent sub-sections.

#### 4.2.1. Density

**Figure 8:**
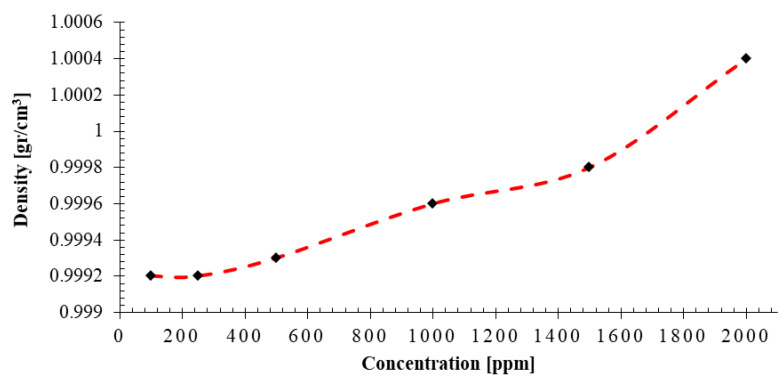
Density of the nanofluids prepared with different concentrations of the novel NC. There is a linear relationship between the density and concentration of the nanofluids.

The density of the prepared nanofluids at different concentrations is presented in Figure 8. Density of the prepared nanofluids are on the range of 0.9992 and 1.0004 g/cm^3^. As one can see from Figure 8, the solution density increases with increase in concentration of the NC. An increase in the nanofluid solution density can lessen the advectve flow instabilty effects caused by hihg mobility ratio in some petroleum reservoirs.

#### 4.2.2. pH

**Figure 9:**
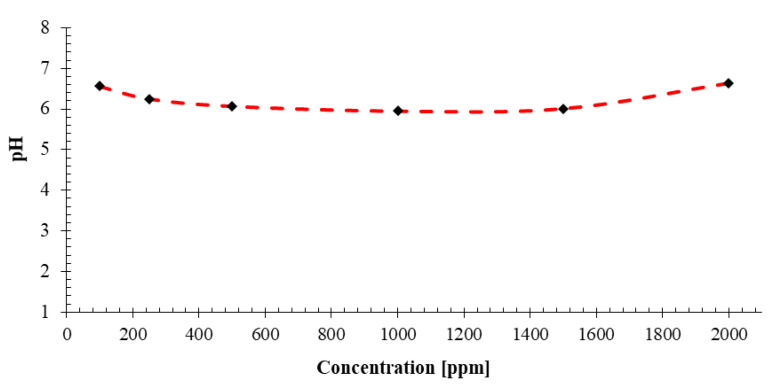
pH values determined for the nanofluids prepared using different concentrations of the novel NC (100–2000 ppm). pH of the prepared nanofluids is almost insensitive to changes in concentration of the novel NC.

Variations in the pH values of the prepared nanofluid for concentrations of 100–2000 ppm of the novel NC is presented in Figure 9. As shown in Figure 9, the pH values of the prepared nanofluids at different concentrations ranges from 5.95 to 6.64. It seems that NC concentration does not affect the pH of the nanofluids much.

#### 4.2.3. Viscosity

**Figure 10:**
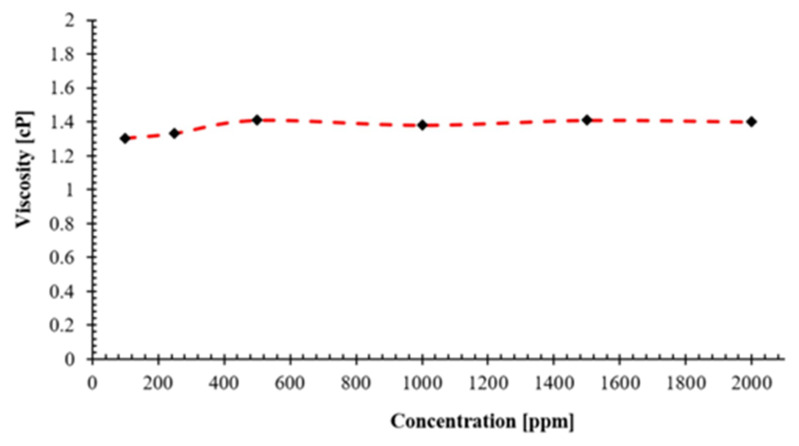
Viscosity values determined for the nanofluids prepared using different concentrations of the novel NC (100–2000 ppm). The viscosity of the prepared nanofluids is almost constant at different concentrations of the novel NC.

Figure 10 shows the viscosity values determined for the prepared nanofluids versus different concentrations of the novel NC (100–2000 ppm). The viscosity the nanofluid ranges from 1.3 cP to 1.4 cP and it changes very little with increase in concentration of the novel NC.

#### 4.2.4. Conductivity

**Figure 11:**
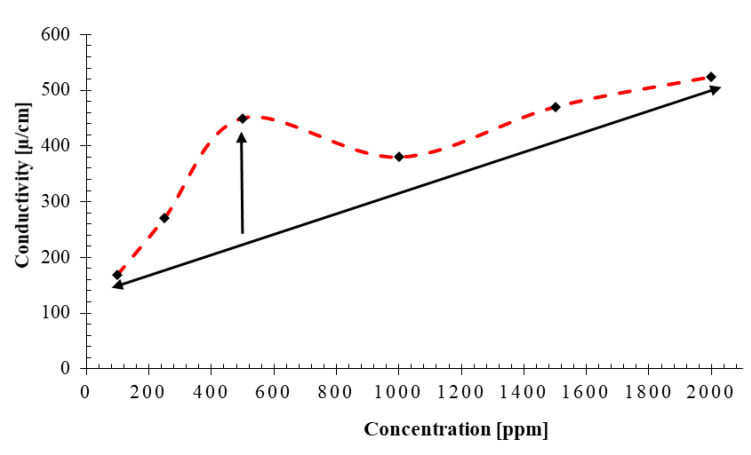
Conductivity of the prepared nanofluids. The minimum and maximum conductivity of 169 µS/cm and 524 µS/cm are recorded for NC concentrations of 100 ppm and 2000 ppm, respectively. An abnormally high conductivity of 480 µS/cm is recorded for the nanofluid with NC concentration of 500 ppm.

The electrical conductivity versus concentration for the prepared nanofluids is presented in Figure 11. As one can see from the Figure 11, the minimum and maximum conductivity of 169 μS/cm and 524 μS/cm are recoreded for concentrations of 100 ppm and 2000 ppm, respectively. However, an abnormally high conductivity of 480 μS/cm is recorded for the nanofluid with NC concentration of 500 ppm which matches the critical micelle concentration or CMC point determined. Depending on the type of material used, this anomaly can be attributed to release of ions into the environment which leads to an increase in the electrical conductivity at this concentration. This abnormal increase in conductivity of the nanofluid prepared with 500 ppm of the novel NC can be attributed to the increase in net charge effect of the ions and the related electrical double-layer interactions in the environment. Such interactions were notable only in 500 ppm concentration as shown in Figure 11 [62,63].

#### 4.2.5. Zeta Potential

**Figure 12:**
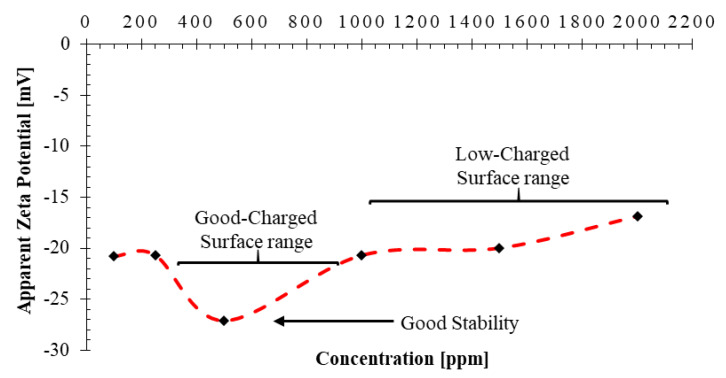
Zeta potential measured for the prepared nanofluids. The measured zeta potential peaks at NC concentration of 500 ppm.

The zeta potential measured for the prepared nanofluids at different concentrations is presented in Figure 12. As can be seen from Figure 12, the measured zeta potential peaks at the concentration of 500 ppm. This increase in zeta potential can be interpreted as an indication to increase in potential difference between stationary layers of the fluid by increase in the concentration of ions (inter-structural charge) attached to the dispersed nanoparticles and the dispersion medium. This can make the prepared nanofluids significantly more stable [64,65,66].

#### 4.2.6. IFT

**Figure 13:**
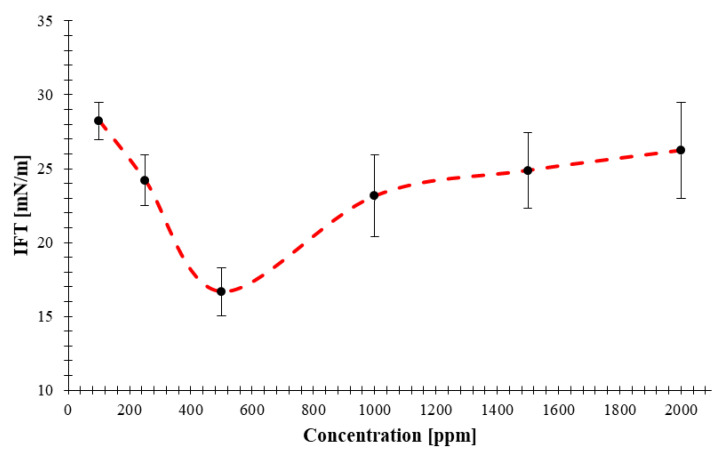
IFT measurement results for the prepared nanofluids.

**Figure 14:**
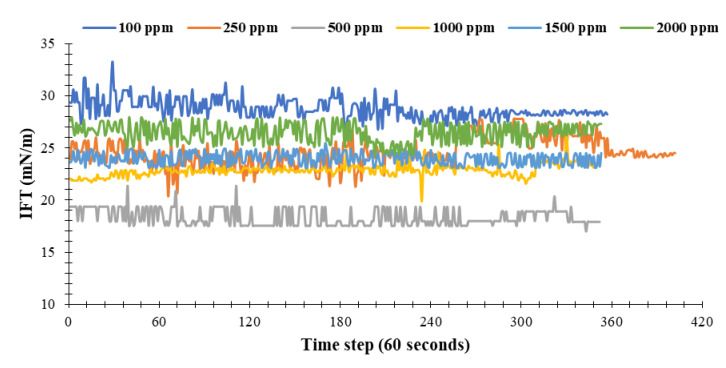
Dynamically measured water−oil interfacial tension over time at ambient pressure (14.7 psi) and temperature (25 °C).

Interfacial tension is one of the main parameters affecting performance of EOR processes. This is because any changes in IFT can affect the amount of capillary number and capillary force in a porous medium. Any changes in theres two parameters can increase the oil recovery from a petroleum reservoir [67,68]. In this section, we examined the effect of nanofluids prepared by using different concentrations of the novel NC. The results of IFT measurement for the prepared nanofluids conducted by using the pendant drop method are shown in Figure 13. The IFT values measured for the nanofluids prepared with concentrations of 100, 250, 500, 1000, 1500, and 2000 ppm of the NC determined as 28, 24, 17, 23, 24, and 26 mN/m, respectively. As can be seen from Figure 13, the largest drop in IFT is observed for the nanofluid prepared with a concentration of 500 ppm. This concentration of nanocomposite can be considered as the optimum concentration for preparation of nanofluids to maximize IFT reduction [69]. The reason for the decrease in IFT at this concentration can be attributed to the increase in conductivity the presence of active ions (charge effect) in the environment [70]. To prove this effect, according to Figure 11 which shows the conductivity of the prepared nanofluids, one can see that conductivity of the nanofluids increases linearly with an increasie in concentration [71,72]. However, at concentration of 500 ppm, the graph shows an anomalous behavior where conductivity increases (increased net charge effect of the ions and the related electrical double-layer interactions in the environment) at this concentration [73,74,75,76]. In addition, according to the result of the zeta potential test, it shows that there is an anomaly at point 500 ppm, which indicates an increase in the degree of stability (related to the net charge effect of the solid particles and the related electrical double-layer interactions) in the environment. IFT of the deionized water and crude oil was measured as 35 mN/m; while, the optimum concentration provides a 17 mN/m reduction in IFT. In other words, the nanofluid prepared by using this novel NC is able to reduce the IFT by 48.5% [76,77,78,79,80,81]. Figure 14 illustrates the measurements of the dynamic IFTs of nanofluids over a period of steps over time (The final and fixed values of IFTs are measured from dynamic values. Each time step is approximately equal to 0.5 s.).

#### 4.2.7. Wettability Measurement

Wettability plays a pivotal role in oil displacement through porous media. In a reservoir, the surface adherence tendency of each fluid differs because there is more than one fluid in the pores. The fluids compete over sticking to the rock surface with different intensities and contact angles which determines the wettability of the rock. Wettability alteration from oil-wet to water-wet is a major mechanism in production of oil by nanofluid in both carbonate and sandstone reservoirs. To evaluate the effect of the novel NC on contact angle, nanofluids with concentrations of 100, 200, 500, 1000, 1500, and 2000 ppm were prepared and then thin sections were prepared using carbonate and sandstone rock samples and were placed in a container containing nanofluid for some time. Then, the contact angles were measured by using the pendant drop method and an image of the oil droplet on the thing sections was captured, as presented in Table 4.

**Figure 15:**
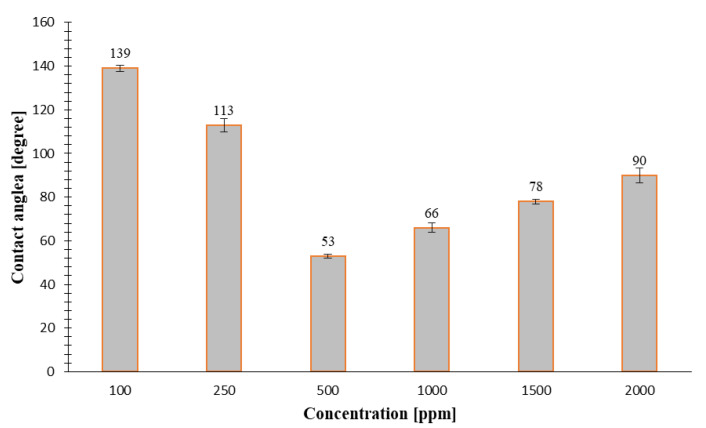
Effect of the prepared nanofluids with different concentrations of the novel NC on wettability (contact angle) alteration in carbonate rock samples.

**Figure 16:**
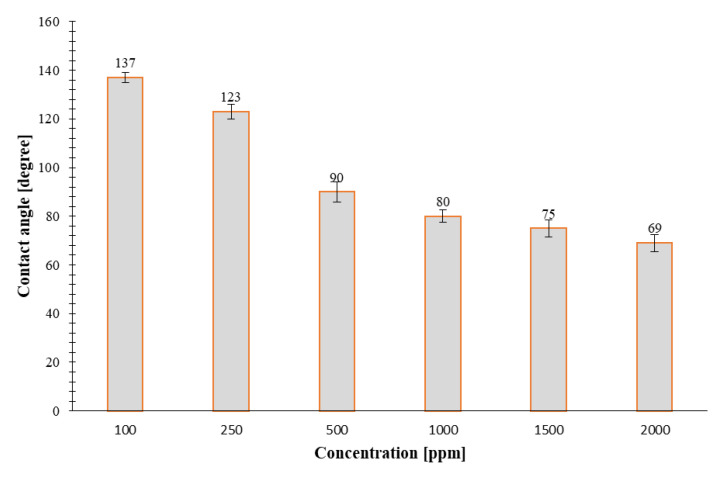
Effect of the prepared nanofluids with different concentrations of the novel NC on wettability (contact angle) of the sandstone rock samples.

Sessile drop method was used to investigate the effect of NC at different concentrations on wettability alteration in both carbonate and sandstone samples as shown in Figure 15 and Figure 16. For concentrations of 100, 250, 500, 1000, 1500, and 2000 ppm a tangential angle of contact of 139°, 113°, 53°, 66°, 78°, and 90° for the carbonate samples and 137°, 123°, 90°, 87°, 75°, and 69° for the sandstone samples was measured using the sessile drop method, respectively. The nanoflud prepared with 500 ppm NC altered the contact angle of carbonate rock sample from 139° to 53° (i.e., to more water-wet) (Figure 15). This can be considered as an optimum concentration to achieve the highest weattability alteration of initial wettability conditions of carbonates towards a more water wet state. In sandstone rock samples, the rock wettability changes towards a more water wet state with increase in concentration of the NC used for preparation of the nanofluids (from 137° to 69°).

The effect of nanofluids prepared by using different concentrations of the novel NC on contact angle and the consequent wettability alteration is far greater than its effect on surface tension. This can be further elaborated by examining the concept of structural separation pressure [82]. When nanofluid enters the pore space then it forms a thin liquid film on the rock surface. The presence of nanoparticles exters a pressure on the thin liquid film or creates a pressure gradient across the thinn liquid film known as separation pressure or structural disjoining pressure. This leads to an increase in entropy and mobility of the nanoparticles in the porous medium. As the entropy increases, separation pressure (disjoining pressure) between the oil and the rock surface separates the oil droplets and increases the surface hydrophilicity (i.e., altering the wettability towards a more water wet state). However, such alterations are controlled by size of the nanoparticles, surface chemistry of the rock, and salinity of interpore space [83,84,85,86,87].

Although, economic considerations are important for successful field application and commercialization of any additives used in EOR processes. Nevertheless, the major cost for this novel NC is the cost of synthesis and mass production. As discussed earlier in this section, improvements in parameters controlling EOR processes by using this novel nanocomposite (CeO_2_@Nanoclay) was achieved by using a very small concentration (500 ppm) of the NC. At this concentration, the nanofluids prepared are capable of reducing the IFT and improving the rock surface wettability in both carbonate and sandstone reservoir rocks which can lead into a notable increase in oil recovery factor from petroleum reservoirs.

After a thorough characterization of the newly synthesized nanocomposite and investigation of its effects on IFT reduction and wettability alteration, additional research work is needed to shed light on unknown aspects of its application for EOR processes. Core flooding experiments are currently underway to assess the performance of the EOR procedure with the newly synthesized nanocomposite. In addition, different combinations of smart water and this nanoclay are being studied to investigate the possibility of combining the nanocomposite with other EOR approaches as hybrid EOR methods. The future research work will focus on: Further understating of mechanism of recovery with the new nanocomposite. Assessment of possible environmental impacts of the nanoclay in the case of its extensive use. Investigation of the requirement or characteristics of the pore size distribution to use this nanocomposite. Simultaneous application of this nanocomposite with other chemical EOR agents such as different surfactants. Study of interaction between this nanocomposite with the PVT of the hydrocarbon system.

## 5. Summary and Conclusions

In this research work, first a novel CeO_2_@nanoclay nanocomposite was synthesied using *Capsicum frutescens* extract and characterised by using various analytical methods including SEM, XRD, FTIR, TGA, EDS and EDS MAP to investigate its surface morphology, crystalline phases, and functional groups. Nanofluids were then prepared with concentration of 100, 250, 500, 1000, 1500, and 2000 ppm of the novel NC. In the next step, different characteristics of the prepared nanofluids such as density, pH, viscosity, conductivity, IFT, zeta potential, and contact angle as factors affecting performance of EOR processes to determine optimum concentration of the NC for EOR purposes in selected carbonate and sandstone reservoir rock samples. The main findings of this research wrok can be summarized as follows:

(1) Based on the XRD test results, the morphology of the novel CeO_2_@nanoclay NC is calcined and CeO_2_ is the predominant phase in the substance. The governed peaks at angles of 29.15°, 48.05°, and 56.9° are related to CeO_2_ crystallography planes of (111), (220), and (311), respectively. A cubic fluorite structure was also identified for CeO_2_ using the standard data.
(2) The infrared spectrum (FTIR) of the synthesized NC (CeO_2_@nanoclay) was recorded in the wavenumber range of 400–4000 cm^−1^. The large broad bands at 3409 cm^−1^ and 3626 cm^−1^ are attributed to the stretching vibration of O–H in OH groups. The absorption peaks of approximately 1641 cm^−1^ were attributed to the bending vibration of the C=C stretching.
(3) FESEM revealed that the surface morphology of the NC has a layer sheet morphology of CeO_2_/SiO_2_ nanocomposite and the particle sizes are approximately 20 to 26 nm.
(4) TGA analysis results shows that the novel NC has a low weight loss (8.21%) and high stability at 90 °C which is a typical upper bound temperature in petroleum reservoirs.
(5) EDS analysis confirm presence of O, Si, and Ce in the novel NC. Atomic and weight analysis of O, Si, and Ce of the NC are determined as (79.47%, 20.32%, and 0.225%) and (67.91%, 30.48%, and 1.61%), respectively.
(6) Density of the prepared nanofluids are on the range of 0.9992 and 1.0004 g/cm^3^. The solution density increases with increase in concentration of the NC. The pH values of the prepared nanofluids at different concentrations ranges from 5.95 to 6.64. It seems that NC concentration does not affect the pH of the nanofluids much. The viscosity the nanofluid ranges from 1.3 cP to 1.4 cP and it changes very little with increase in concentration of the novel NC.
(7) The minimum and maximum conductivity of 169 μS/cm and 524 μS/cm are recoreded for concentrations of 100 ppm and 2000 ppm, respectively. An abnormally high conductivity of 480 μS/cm is recorded for the nanofluid with NC concentration of 500 ppm which matches the critical micelle concentration or CMC point determined. The measured zeta potential peaks at the concentration of 500 ppm which is a sign of stabilty of the nanofluid.
(8) The IFT values measured for the nanofluids prepared with concentrations of 100, 250, 500, 1000, 1500, and 2000 ppm of the NC determined as 28, 24, 17, 23, 24, and 26 mN/m, respectively. The largest drop in IFT is observed for the nanofluid prepared with a concentration of 500 ppm. This concentration of nanocomposite can be considered as the optimum concentration for preparation of nanofluids to maximize IFT reduction. IFT of the deionized water and crude oil was measured as 35 mN/m; while, the optimum concentration provides a 17 mN/m reduction in IFT. In other words, the nanofluid prepared by using this novel NC is able to reduce the IFT by 48.5%.
(9) For concentrations of 100, 250, 500, 1000, 1500, and 2000 ppm a tangential angle of contact of 139°, 113°, 53°, 66°, 78°, and 90° for the carbonate samples and 137°, 123°, 90°, 87°, 75°, and 69° for the sandstone samples was measured using the sessile drop method, respectively. The nanoflud prepared with 500 ppm NC altered the contact angle of carbonate rock sample from 139° to 53° (i.e., to more water-wet). This can be considered as an optimum concentration to achieve the highest weattability alteration of initial wettability conditions of carbonates towards a more water wet state. In sandstone rock samples, the rock wettability changes towards a more water wet state with increase in concentration of the NC used for preparation of the nanofluids (from 137° to 69°).

## Figures and Tables

**Table 1:** A summary review of some previous studies reported on application of nanoparticle in EOR processes.

Year [Reference]	Author	Material	Result
2001 [30]	Alomair et al.	SiO_2_, Al_2_O_3_, and NiO	SiO_2_ and NiO NPs have the highest and lowest effect on IFT reduction, respectively. The low IFT reduction by NiO NPs is attributed to the high adsorption of the NiO NPs on the rock surface.
2008 [31]	Jiang et al.	Fe_2_O_3_/Fe_3_O_4_	Iron Oxide can increase the RF up to 82.5% under laboratory conditions.
2009 [32]	Shah et al.	CuO NP	Can increase oil recovery from heavy oil reservoirs.
2010 [33]	Yuan et al.	Polymeric microspheres and nanospheres	Can reduce the water cut and increase the sweep efficiency and oil recovery.
2012 [34]	Bagherzadeh et al.	MgO, Al_2_O_3_, CeO_2_, CNT, SiO_2_, TiO_2_, CaCO_3_, ZrO_2_	Applied in both carbonate and sandstone core samples. CaCo_3_ and SiO_2_ generally had the highest recovery factor. MgO nanoparticles can prevent fines migration.
2013 [18]	Hendraningrat et al.	Al_2_O_3_, TiO_2_, SiO_2_	Nanofluids containing Al_2_O_3_ can change the wettability of sandstone cores from oil-wet to water-wet. TiO_2_ can alter the wettability of quartz plate to water-wet better than Al_2_O_3_ and SiO_2_.
2015 [34]	Roustaei et al.	Nanofluid with SiO_2_ NPs	SiO_2_ NPs can be considered as potential wettability modifiers.
2014 [35]	Mohsen Seid Mohammadi et al.	γ-AL_2_O_3_	ϒ-AL_2_O_3_ adsorption on calcite surface can alter the wettability from an oil-wet to a water-wet and increase the RF from 11.25% to 65%.
2015 [36]	Adel et al.	Al_2_O_3_ and SiO_2_ and nanofluid	IFT reduction occurs when each of the NPs are added to the brine. Al_2_O_3_ nanofluid had higher IFT compared to SiO_2_. Hence, Al_2_O_3_ has a lower potential to produce more oil compared to SiO_2_.
2015 [37]	Maghzi et al.	SiO_2_ NPs	SiO_2_ NPs can change the wettability from oil-wet to water-wet irrespective of the oil viscosity.
2016 [38]	Kapusta et al.	SiO_2_	SiO_2_ can alter the wettability of an oil-wet and mixed-wet calcite layer. The NP adsorption occurs on the surface and it is irreversible.
2016 [19]	Assef et al.	MgO on LoSal water flooding	Adding MgO NPs into a low salinity solution leads to more positive zeta potential in the porous medium.
2019 [39]	Bahraminejad et al.	CuO/TiO_2_/PAM	This nanocomposite can reduced water−oil IFT by about 46% at a low concentration of 200 ppm and can change the wettability of carbonate rocks from oil-wet to strongly water-wet (from 151° to 14.7°).
2020 [40]	Nowrouzi et al.	TiO_2_, MgO, γ-Al_2_O_3_	TiO_2_ NPs can change the wettability from oil-wet to water-wet and increase the recovery factor up to 75.93%.

**Table 2:** Properties of the crude oil used in this study.

Component	C_1_	C_2_	C_3_	iC_4_	nC_4_	iC_5_	nC_5_	C_6_	C_7_	C_8_	C_9_	C_10_	C_11_	C_12_^+^	Total
Molar %	0.00	0.08	0.73	0.72	2.22	1.10	1.10	8.66	9.32	6.60	7.14	5.36	5.01	51.96	100.0
Molecular weight (MW) = 247 Molecular weight of C+^12^ = 380 Specific gravity of C_12_^+^@ 15.55 °C = 0.9369 Saturation pressure of reservoir fluid @ 60.6 °C = 14.04 MPa									SARA analysis of oil sample						
									Saturates (%)		Aromatics (%)		Resins (%)		Asphaltene (%)
									45		32		5		8

**Table 3:** Properties of the nanofluid prepared with different concentrations of the novel NC.

NC Concentration [ppm]	Zeta Potential (mv)	pH	Viscosity (cP)	Conductivity (µs/cm)	Density (g/cm^3^)	IFT (mN/m)
100	−20.8	6.56	1.3	169	0.9992	28
250	−20.7	6.25	1.33	270	0.9992	24
500	−27.1	6.07	1.41	480	0.9993	17
1000	−20.7	5.95	1.38	380	0.9996	23
1500	−20	6.01	1.41	470	0.9998	24
2000	−16.9	6.64	1.40	524	1.0004	26

**Table 4:** The effect of the nanofluids prepared with different concentrations of the NC on wettability (contact angle or CA) of the tested carbonate and sandstone samples. The contact angles were measured by using the pendant drop method and an image of the oil droplet on the thing sections were captured.

Nano Fluid Concentration (ppm)	Initial CA	Final CA of Carbonate	Final CA of Sandstone
100	Carbonate 150	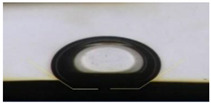 139	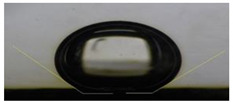 137
	Sandstone 140		
250	Carbonate 150	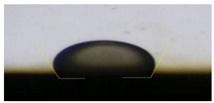 113	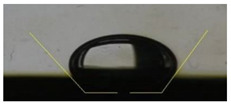 123
	Sandstone 140		
500	Carbonate 150	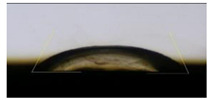 53	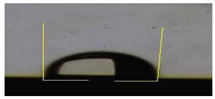 90
	Sandstone 140		
1000	Carbonate 150	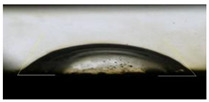 66	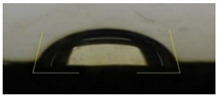 87
	Sandstone 140		
1500	Carbonate 150	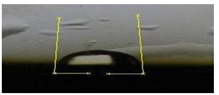 78	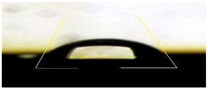 75
	Sandstone 140		
2000	Carbonate 150	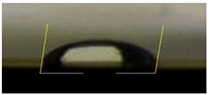 90	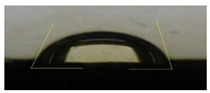 69

## References

[1] IEA (2019). World Energy Outlook 2019.

[2] Mohammadi M., Riahi S. (2020). Experimental investigation of water incompatibility and rock/fluid and fluid/fluid interactions in the absence and presence of scale inhibitors. SPE J..

[3] Green D.W., Willhite G.P. (1998). Enhanced Oil Recovery.

[4] Sinaga F.T.H., Napitupulu F.H., Nur T.B. (2020). Hydrogen gas production simulation utilizing empty fruit bunch of oil palm pyrolysis unit by steam methane reforming process. J. Phys. Conf. Ser..

[5] Mohanty K., Chandrasekhar S. Wettability alteration with brine composition in high temperature carbonate reservoirs. Proceedings of the SPE Annual Technical Conference and Exhibition.

[6] Naik S., You Z., Bedrikovetsky P. (2018). Productivity index enhancement by wettability alteration in two-phase compressible flows. J. Nat. Gas Sci. Eng..

[7] (2015). Instrumental characterization of montmorillonite Clay by FT-IR and XRD from JKUAT farm, in the Republic of Kenya. Chem. Mat. Res..

[8] Zhang P., Tweheyo A.M.T., Austad T. (2006). Wettability alteration and improved oil recovery in chalk: The effect of calcium in the presence of sulfate. Energy Fuels.

[9] Negin C., Ali S., Xie Q. (2016). Application of nanotechnology for enhancing oil recovery—A review. Petroleum.

[10] Kazemzadeh Y., Parsaei R., Riazi M. (2015). Experimental study of asphaltene precipitation prediction during gas injection to oil reservoirs by interfacial tension measurement. Colloids Surf. A Physicochem. Eng. Asp..

[11] Cheraghian G., Hendraningrat L. (2016). A review on applications of nanotechnology in the enhanced oil recovery part B: Effects of nanoparticles on flooding. Int. Nano Lett..

[12] Lai N., Li S., Liu L., Li Y., Li J., Zhao M. (2017). Synthesis and rheological property of various modified nano-SiO_2_/AM/AA hyperbranched polymers for oil displacement. Russ. J. Appl. Chem..

[13] Gbadamosi A.O., Junin R., Manan M.A., Yekeen N., Junin R., Oseh J.O. (2018). Recent advances and prospects in polymeric nanofluids application for enhanced oil recovery. J. Ind. Eng. Chem..

[14] Yang Y., Cheng T., Wu H., You Z., Shang D., Hou J. (2020). Enhanced oil recovery using oleic acid modified titania nanofluids: Underlying mechanisms and oil-displacement performance. Energy Fuels.

[15] Manshad A.K., Rezaei M., Moradi S., Nowrouzi I., Mohammadi A.H. (2017). Wettability alteration and interfacial tension (IFT) reduction in enhanced oil recovery (EOR) process by ionic liquid flooding. J. Mol. Liq..

[16] Khademolhosseini R., Jafari A.J., Shabani M. (2015). Micro scale investigation of enhanced oil recovery using nano/bio materials. Procedia Mater. Sci..

[17] Bera A., Belhaj H. (2016). Application of nanotechnology by means of nanoparticles and nano-dispersions in oil recovery—A comprehensive review. J. Nat. Gas Sci. Eng..

[18] Hendraningrat L., Torsaeter O. (2014). Metal oxide-based nanoparticles: Revealing their potential to enhance oil recovery in different wettability systems. Appl. Nanosci..

[19] Assef Y., Arab D., Pourafshary P. (2014). Application of nanofluid to control fines migration to improve the performance of low salinity water flooding and alkaline flooding. J. Pet. Sci. Eng..

[20] Lager A., Webb K.J., Collins I.R., Richmond D.M. (2008). LoSal enhanced oil recovery: Evidence of enhanced oil recovery at the reservoir scale. Proceedings of the SPE Symposium on Improved Oil Recovery, Tulsa, Oklahoma, USA, 20–23 April 2008.

[21] Sun X., Zhang Y., Chen G., Gai Z. (2017). Application of nanoparticles in enhanced oil recovery: A critical review of recent progress. Energies.

[22] Shamilov V., Babayev E., Kalbaliyeva E., Shamilov F. (2017). Polymer nanocomposites for enhanced oil recovery. Mater. Today: Proc..

[23] Zheng C., Cheng Y., Wei Q., Li X., Zhang Z. (2017). Suspension of surface-modified nano-SiO_2_ in partially hydrolyzed aqueous solution of polyacrylamide for enhanced oil recovery. Colloids Surf. A Physicochem. Eng. Asp..

[24] Yousefvand H., Jafari A.J. (2015). Enhanced Oil Recovery Using Polymer/nanosilica. Procedia Mater. Sci..

[25] Luo P., Luo W., Li S. (2017). Effectiveness of miscible and immiscible gas flooding in recovering tight oil from Bakken reservoirs in Saskatchewan, Canada. Fuel.

[26] Bennetzen M.V., Mogensen K. Novel applications of nanoparticles for future enhanced oil recovery. Proceedings of the International Petroleum Technology Conference.

[27] Moghaddam R.N., Bahramian A., Fakhroueian Z., Karimi A., Arya S. (2015). Comparative study of using nanoparticles for enhanced oil recovery: Wettability alteration of carbonate rocks. Energy Fuels.

[28] Cheraghian G., Nezhad S.S.K., Kamari M., Hemmati M., Masihi M., Bazgir S. (2014). Effect of nanoclay on improved rheology properties of polyacrylamide solutions used in enhanced oil recovery. J. Pet. Explor. Prod. Technol..

[29] Delshad M., Kim D.H., Magbagbeola O.A., Huh C., Pope G.A., Tarahhom F. Mechanistic interpretation and utilization of viscoelastic behavior of polymer solutions for improved polymer-flood efficiency. Proceedings of the SPE Symposium on Improved Oil Recovery.

[30] Alomair O.A., Matar K.M., AlSaeed Y.H. Nanofluids application for heavy oil recovery. Proceedings of the SPE Asia Pacific Oil & Gas Conference and Exhibition.

[31] Wu W., He Q., Jiang C. (2008). Magnetic iron oxide nanoparticles: Synthesis and surface functionalization strategies. Nanoscale Res. Lett..

[32] Shah R.D. Application of nanoparticle saturated injectant gases for EOR of heavy oils. Proceedings of the SPE Annual Technical Conference and Exhibition.

[33] Yuan B., Moghanloo R.G., Zheng D. Enhanced oil recovery by combined nanofluid and low salinity Water flooding in multi-layer heterogeneous reservoirs. Proceedings of the SPE Annual Technical Conference and Exhibition.

[34] Roustaei A., Moghadasi J., Bagherzadeh H., Shahrabadi A. An experimental investigation of polysilicon nanoparticles’ recovery efficiencies through changes in interfacial tension and wettability alteration. Proceedings of the SPE International Oilfield Nanotechnology Conference and Exhibition.

[35] Seid Mohammadi M., Moghadasi J., Naseri S. (2014). An experimental investigation of wettability alteration in carbonate reservoir using γ-Al_2_O_3_ nanoparticles. Iran. J. Oil Gas Sci. Technol..

[36] Ragab A.M.S., Hannora A.E. A Comparative investigation of nano particle effects for improved oil recovery—Experimental work. Proceedings of the SPE Kuwait Oil & Gas Show and Conference.

[37] Maghzi A., Kharrat R., Mohebbi A., Ghazanfari M.H. (2014). The impact of silica nanoparticles on the performance of polymer solution in presence of salts in polymer flooding for heavy oil recovery. Fuel.

[38] Kapusta S., Balzano L., Te Riele P. (2012). Nanotechnology applications in oil and gas exploration and production. Proceedings of the IPTC 2011: International Petroleum Technology Conference, Bangkok, Thailand, 7 February 2012.

[39] Bahraminejad H., Khaksar Manshad A., Riazi M., Ali J.A., Sajadi S.M., Keshavarz A. (2019). CuO/TiO_2_/PAM as a novel introduced hybrid agent for water—Oil interfacial tension and wettability optimization in chemical enhanced oil recovery. Energy Fuels.

[40] Nowrouzi I., Manshad A.K., Mohammadi A.H. (2020). Effects of TiO_2_, MgO and γ-Al_2_O_3_ nano-particles on wettability alteration and oil production under carbonated nano-fluid imbibition in carbonate oil reservoirs. Fuel.

[41] Trache D., Thakur V.K., Boukherroub R. (2020). Cellulose nanocrystals/graphene hybrids—A promising new class of materials for advanced applications. Nanomaterials.

[42] Ateş B., Koytepe S., Ulu A., Gurses C., Thakur V.K. (2020). Chemistry, structures, and advanced applications of nanocomposites from biorenewable resources. Chem. Rev..

[43] Long Y., Wang R., Zhu B., Huang X., Leng Z., Chen L., Song F. (2019). Enhanced oil recovery by a suspension of core-shell polymeric nanoparticles in heterogeneous low-permeability oil reservoirs. Nanomaterials.

[44] Aadland R.C., Akarri S., Heggset E.B., Syverud K., Torsæter O. (2020). A core flood and microfluidics investigation of nanocellulose as a chemical additive to water flooding for EOR. Nanomaterials.

[45] Mehrabianfar P., Malmir P., Soulgani B.S., Hashemi A. (2020). Study on the optimization of the performance of preformed particle gel (PPG) on the isolation of high permeable zone. J. Pet. Sci. Eng..

[46] Bila A., Stensen J.A., Torsæter O. (2019). Experimental investigation of polymer-coated silica nanoparticles for enhanced oil recovery. Nanomaterials.

[47] Franco C.A., Giraldo L.J., Candela C.H., Bernal K.M., Villamil F., Montes D., Lopera S.H., Franco C.A., Cortés F.B. (2020). Design and tuning of nanofluids applied to chemical enhanced oil recovery based on the surfactant–nanoparticle–brine interaction: From laboratory experiments to oil field application. Nanomaterials.

[48] Li S., Ng Y.H., Lau H.C., Torsæter O., Stubbs L.P. (2020). Experimental investigation of stability of silica nanoparticles at reservoir conditions for enhanced oil recovery applications. Nanomaterials.

[49] Adil M., Lee K., Zaid H.M., Manaka T. (2020). Role of phase dependent dielectric properties of alumina nanoparticles in electromagnetic assisted enhanced oil recovery. Nanomaterials.

[50] Rezaei A., Abdollahi H., Derikvand Z., Hemmati-Sarapardeh A., Mosavi A., Nabipour N. (2020). Insights into the effects of pore size distribution on the flowing behavior of carbonate rocks: Linking a nano-based enhanced oil recovery method to rock typing. Nanomaterials.

[51] Medina O.E., Gallego J., Arias-Madrid D., Cortés F.B., Franco C.A. (2019). Optimization of the load of transition metal oxides (Fe_2_O_3_, Co_3_O_4_, NiO and/or PdO) onto CeO_2_ nanoparticles in catalytic steam decomposition of n-C7 asphaltenes at low temperatures. Nanomaterials.

[52] Medina O.E., Gallego J., Restrepo L.G., Cortés F.B., Franco C.A. (2019). Influence of the Ce_4+_/Ce_3+_ Redox-couple on the cyclic regeneration for adsorptive and catalytic performance of NiO-PdO/CeO_2±δ_ nanoparticles for n-C_7_ asphaltene steam gasification. Nanomaterials.

[53] Pérez-Robles S., Matute C.A., Lara J.R., Lopera S.H., Cortés F.B., Franco C.A. (2019). Effect of nanoparticles with different chemical nature on the stability and rheology of acrylamide sodium acrylate copolymer/chromium (III) acetate gel for conformance control operations. Nanomaterials.

[54] Di Sacco F., Pucci A., Raffa P. (2019). Versatile multi-functional block copolymers made by atom transfer radical polymerization and post-synthetic modification: Switching from volatile organic compound sensors to polymeric surfactants for water rheology control via hydrolysis. Nanomaterials.

[55] Kamaei E., Manshad A.K., Shadizadeh S.R., Ali J.A., Keshavarz A. (2019). Effect of the wettability alteration on the cementation factor of carbonate rocks using Henna extract. Materialia.

[56] Eslahati M., Mehrabianfar P., Isari A.A., Bahraminejad H., Manshad A.K., Keshavarz A. (2020). Experimental investigation of Alfalfa natural surfactant and synergistic effects of Ca^2+^, Mg^2+^, and SO_4_^2−^ ions for EOR applications: Interfacial tension optimization, wettability alteration and imbibition studies. J. Mol. Liq..

[57] Aadland R.C., Jakobsen T.D., Heggset E.B., Torsæter O., Simon S., Paso K.G., Syverud K., Torsæter O. (2019). High temperature core flood investigation of nanocellulose as a green additive for enhanced oil recovery. Nanomaterials.

[58] Rueda E., Akarri S., Torsæter O., Moreno R.B. (2020). Experimental investigation of the effect of adding nanoparticles to polymer flooding in water-wet micromodels. Nanomaterials.

[59] Dong M.W. (2000). Instruments & applications. How hot is that pepper? Quantifying capsaicinoids with chromatography. Todays Chem. Work.

[60] Nwokem C.O. (2017). Determination of capsaicin content and pungency level of five different Fresh and Dried Chilli Peppers. N. Y. Sci. J..

[61] Bello I., Boboye B.E., Akinyosoye F.A. (2015). Phytochemical screening and antibacterial properties of selected Nigerian long pepper (Capsicum frutescens) fruits. Afr. J. Microbiol. Res..

[62] Khan N., Aziz K. (2015). Comparative study of sports competitive anxiety and sports achievement motivation between basketball players and all India intervarsity running events athletes. Int. J. Modern Chem. Appl. Sci..

[63] Kumar A., Dixit C.K. (2017). Methods for characterization of nanoparticles. Advances in Nanomedicine for the Delivery of Therapeutic Nucleic Acids.

[64] Farahmandjou M., Zarinkamar M., Firoozabadi T.P. (2016). Synthesis of Cerium Oxide (CeO_2_) nanoparticles using simple CO-precipitation method. Rev. Mex. de Física.

[65] Tok A.I.Y., Boey F.Y.C., Dong Z., Sun X. (2007). Hydrothermal synthesis of CeO_2_ nano-particles. J. Mater. Process. Technol..

[66] Vieillard P., Tajeddine L., Gailhanou H., Blanc P., Lassin A., Gaboreau S. (2015). Thermo-analytical techniques on MX-80 montmorillonite: A way to know the behavior of water and its thermodynamic properties during hydration–dehydration processes. Pharm. Anal. Acta.

[67] Abramov A., Keshavarz A., Iglauer S. (2019). Wettability of fully hydroxylated and alkylated (001) α-quartz surface in carbon dioxide atmosphere. J. Phys. Chem. C.

[68] Fu A., Gu W., Larabell C., Alivisatos A.P. (2005). Semiconductor nanocrystals for biological imaging. Curr. Opin. Neurobiol..

[69] Nowakowski P., Villain S., Aguir K., Guerin J., Kopia A., Kusinski J., Guinneton F., Gavarri J. (2010). Microstructure and electrical properties of RuO_2_–CeO_2_ composite thin films. Thin Solid Films.

[70] Kumar E., Selvarajan P., Muthuraj D. (2012). Preparation and characterization of polyaniline/cerium dioxide (CeO_2_) nanocomposite via in situ polymerization. J. Mater. Sci..

[71] Harlem G., Picaud F., Giradet C., Micheau O. (2019). Nano-Carriers for Drug Delivery: Nanoscience and Nanotechnology in Drug Delivery.

[72] Shnoudeh A.J., Hamad I., Abdo R.W., Qadumii L., Jaber A.Y., Surchi H.S., Alkelany S.Z. (2019). Synthesis, characterization, and applications of metal nanoparticles. Biomaterials and Bionanotechnology.

[73] Jalil R.R., Hussein H. (2019). Influence of nano fluid on interfacial tension oil/water and wettability alteration of limestone. IOP Conf. Ser. Mater. Sci. Eng..

[74] Nowrouzi I., Manshad A.K., Mohammadi A.H. (2018). Effects of dissolved binary ionic compounds and different densities of brine on interfacial tension (IFT), wettability alteration, and contact angle in smart water and carbonated smart water injection processes in carbonate oil reservoirs. J. Mol. Liq..

[75] Chengara A., Nikolov A.D., Wasan D.T., Trokhymchuk A., Henderson D. (2004). Spreading of nanofluids driven by the structural disjoining pressure gradient. J. Colloid Interface Sci..

[76] Zargar G., Arabpour T., Manshad A.K., Ali J.A., Sajadi S.M., Keshavarz A., Mohammadi A.H. (2020). Experimental investigation of the effect of green TiO_2_/Quartz nanocomposite on interfacial tension reduction, wettability alteration, and oil recovery improvement. Fuel.

[77] Ali J., Manshad A.K., Imani I., Sajadi S.M., Keshavarz A. (2020). Greenly synthesized magnetite@ SiO_2_@ xanthan nanocomposites and its application in enhanced oil recovery: IFT reduction and wettability alteration. Arab. J. Sci. Eng..

[78] Ali J.A., Ali J.A., Manshad A.K., Mohammadi A.H. (2018). Recent advances in application of nanotechnology in chemical enhanced oil recovery: Effects of nanoparticles on wettability alteration, interfacial tension reduction, and flooding. Egypt. J. Pet..

[79] Moradi S., Isari A.A., Bachari Z., Mahmoodi H. (2019). Combination of a new natural surfactant and smart water injection for enhanced oil recovery in carbonate rock: Synergic impacts of active ions and natural surfactant concentration. J. Pet. Sci. Eng..

[80] Nowrouzi I., Manshad A.K., Mohammadi A.H. (2019). Effects of concentration and size of TiO_2_ nano-particles on the performance of smart water in wettability alteration and oil production under spontaneous imbibition. J. Pet. Sci. Eng..

[81] Ali J.A., Kolo K., Manshad A.K., Stephen K., Keshavarz A. (2018). Modification of LoSal water performance in reducing interfacial tension using green ZnO/SiO_2_ nanocomposite coated by xanthan. Appl. Nanosci..

[82] Najimi S., Nowrouzi I., Manshad A.K., Farsangi M.H., Hezave A.Z., Ali J.A., Keshavarz A., Mohammadi A.H. (2019). Investigating the effect of [C8Py][Cl] and [C18Py][Cl] ionic liquids on the water/oil interfacial tension by considering Taguchi method. J. Pet. Explor. Prod. Technol..

[83] Asl H.F., Zargar G., Manshad A.K., Takassi M.A., Ali J.A., Keshavarz A. (2020). Effect of SiO_2_ nanoparticles on the performance of L-Arg and L-Cys surfactants for enhanced oil recovery in carbonate porous media. J. Mol. Liq..

[84] Asl H.F., Zargar G., Manshad A.K., Takassi M.A., Ali J.A., Keshavarz A. (2019). Experimental investigation into l-Arg and l-Cys eco-friendly surfactants in enhanced oil recovery by considering IFT reduction and wettability alteration. Pet. Sci..

[85] Manshad A., Ali J., Imani I., Tayeb N., Keshavarz A. (2020). Green synthesize of CuO@ Fe_3_O_4_@ xantan nanocomposites and its application in enhanced oil recovery by considering IFT and wettability behaviors. Micro Nano Lett..

[86] Al-Anssari S., Barifcani A., Keshavarz A., Iglauer S. (2018). Impact of nanoparticles on the CO_2_-brine interfacial tension at high pressure and temperature. J. Colloid Interface Sci..

[87] Alizadeh A.H., Keshavarz A., Haghighi M. (2007). Flow rate effect on two-phase relative permeability in Iranian carbonate rocks. Proceedings of the SPE Middle East Oil and Gas Show and Conference, Manama, Bahrain, 11–14 March 2007.

